# Multi-omics analysis reveals prognostic and therapeutic value of cuproptosis-related lncRNAs in oral squamous cell carcinoma

**DOI:** 10.3389/fgene.2022.984911

**Published:** 2022-08-15

**Authors:** Xiaoguang Li, Wenbin Zhou, Chang Zhu, Jiechen Liu, Zedong Ming, Cong Ma, Qing Li

**Affiliations:** ^1^ Department of Stomatology, Shandong Provincial Hospital Affiliated to Shandong First Medical University, Jinan, China; ^2^ School and Hospital of Stomatology, Cheeloo College of Medicine, Shandong University, Jinnan, China; ^3^ Shandong Key Laboratory of Oral Tissue Regeneration, Jinnan, China; ^4^ Shandong Engineering Laboratory for Dental Materials and Oral Tissue Regeneration, Jinan, China

**Keywords:** oral squamous cell carcinoma, cuproptosis, signature, prognosis, immunotherapy

## Abstract

**Background:** Extensive research revealed copper and lncRNA can regulate tumor progression. Additionally, cuproptosis has been proven can cause cell death that may affect the development of tumor. However, there is little research focused on the potential prognostic and therapeutic role of cuproptosis-related lncRNA in OSCC patients.

**Methods:** Data used were for bioinformatics analyses were downloaded from both the TCGA database and GEO database. The R software were used for statistical analysis. Mapping was done using the tool of FigureYa.

**Results:** The signature consist of 7 cuproptosis-related lncRNA was identified through lasso and Cox regression analysis and a nomogram was developed. In addition, we performed genomic analyses including pathway enrichment analysis and mutation analysis between two groups. It was found that OSCC patients were prone to TP53, TTN, FAT1 and NOTCH1 mutations and a difference of mutation analysis between the two groups was significant. Results of TIDE analysis indicating that patients in low risk group were more susceptible to immunotherapy. Accordingly, results of subclass mapping analysis confirmed our findings, which revealed that patients with low riskscore were more likely to respond to immunotherapy.

**Conclusion:** We have successfully identified and validated a novel prognostic signature with a strong independent predictive capacity. And we have found that patients with low riskscore were more susceptible to immunotherapy, especially PD-1 inhibitor therapy.

## Background

Oral squamous cell carcinoma (OSCC) is one of the most common tumors in head and neck with the highest degree of malignancy, with more than 370,000 new cases diagnosed and 177,000 deaths worldwide (Sung et al., 2021). Epidemiological studies showed that OSCC is more prevalent in men, partly due to factors such as alcoholism and smoking (Warnakulasuriya, 2009). Despite improvements in imaging technologies, surgical methods, radiation treatment, and chemotherapy, there has been no significant improvement in the 5-year survival rate of OSCC patients (Kumar et al., 2016; Zanoni et al., 2019). Worse still, many OSCC patients still battle with the terrible side effects even after receiving treatment, including depression, nutritional deficiencies and damage of the patient’s appearance and ability to complete daily activities (Parke et al., 2022). Currently, histological characteristics are the basis for vast majority of the clinical prediction signatures. Therefore, it is essential to find more effective targets that accurately predict the prognosis of OSCC in order to enhance clinical diagnosis and patient treatment.

Copper (CU), a necessary nutrient for all living organisms, functions as a cofactor in numerous metabolic enzymes and plays an essential role in diverse fundamental biological processes (Grubman and White, 2014). In recent research, it was found that cancer patients had much higher copper levels in their blood and tumor tissues compared to healthy controls (Blockhuys et al., 2017). A recent study proposed a novel form of copper-induced cell death, which is defined cuproptosis, demonstrating that excessive intracellular copper induced proteotoxic stress leading to cell death (Tsvetkov et al., 2022). However, there are few research focused on the cuproptosis and its biological functions in OSCC. Therefore, whether copper-induced cell death is involved in the occurrence and development of OSCC is worthy of our further study. Meanwhile, we noticed the long noncoding RNA (lncRNA), a well-regulated gene regulator, playing a part in various biological and cellular processes, was closely involved in tumorigenesis and progression of various cancers (Fatica and Bozzoni, 2014). Accordingly, a large amount of literature suggested that lncRNA can be used to evaluate cancer prognosis and guide clinical therapies in various tumors including OSCC. For example, lncRNA HOXA11-AS was highly expressed in OSCC tissues and cells compared with healthy controls, which contributes actively to the development of OSCC (Niu et al., 2020). Additionally, in the AKT/mTOR pathway, lncRNA CASC9 helps promote OSCC progression by stimulating cell proliferation and suppressing autophagy-mediated apoptosis (Yang et al., 2019). Moreover, a novel lncRNA ORAOV1-B that can enhance the invasion and metastasis of OSCC by binding to Hsp90 and activating the NF-κB-TNFα signaling loop (Luo et al., 2021). In light of the importance of cuproptosis and lncRNAs, new approaches to predicting the prognosis of OSCC patients may be possible.

In this study, to understand the potential role of cuproptosis-related lncRNA in OSCC, we systematically performed analyses including cox regression analysis, nomogram analysis, pathway enrichment analysis, mutation analysis and immune analysis. Finally, a cuproptosis-related lncRNA based signature was successfully constructed and validated, which could effectively predict prognosis of OSCC patients. Notably, our findings revealed that patients with low riskscore were more susceptible to immunotherapy including PD-1 inhibitor therapy. By taking these results into account, we will be able to better understand the role of cuproptosis-related lncRNA in OSCC and develop personalized treatments for each patient.

## Methods

### Data acquisition and preprocessing

RNA-sequencing data (row count files) and corresponding clinical information of patients with head and neck squamous cell carcinoma (HNSC) were downloaded from the TCGA database (https://portal.gdc.cancer.gov) and GEO database (https://www.ncbi.nlm.nih.gov/geo). TCGA data with anatomic neoplasm subdivision were alveolar ridge, base of tongue, buccal mucosa, floor of mouth, hard palate, hypopharynx, lip, oral cavity, oral tongue, oropharynx and tonsil (OSCC) served as the training cohort, and GSE42743 data served as the validation cohort. All the data were preprocessed by the following steps: standardized the mRNA expression data, patients with well-annotated clinical follow-up information including survival status and survival time more 30 days were selected, merged the mRNA expression data with the clinical information. All the cuproptosis-related genes (*n* = 13) were collected from the known literature (Tang et al., 2022).

### Construction of cuproptosis-related lncRNA prognostic signature

After preprocessing RNA-sequencing data and corresponding clinical information, a total of 390 OSCC patients in TCGA database were identified as a training cohort, and a total of 206 data in GEO database were identified as a validation cohort. Pearson’s correlation analysis was performed to determine the correlation coefficient between the expression of cuproptosis-related genes and the lncRNAs. Then, lncRNAs we regarded as cuproptosis-related lncRNAs according to the following criteria: | correlation coefficient | >0.4 and *p* value less than 0.001 (*p* < 0.001). Next, the lncRNAs associated with overall survival (OS, the time from registration to death from any cause) time of OSCC patients were identified using univariate Cox regression analysis. These lncRNAs were further screened using the lasso regression analysis based on the “glmnet” R package. Following this, a cuproptosis-related lncRNA prognostic signature was constructed based on the cuproptosis-related lncRNAs which were associated with OS identified by multivariate Cox regression analysis. Importantly, the formula used to calculate the riskscore of OSCC patients as follows:

$$riskscore=\exp\left(cuproptosis-related\;\ln cRNAi\right)*\sum_{i=1}^{n}coef\left(\ln cRNAi\right)$$

In this formula, exp (cuproptosis-related lncRNAi) indicated the expression of these lncRNA, and coef (lncRNAi) indicated the Cox coefficient of the these lncRNAs in the signature.

### Verification of the signature and development of the nomogram

First, we used the ‘maxstat’ R package (maximally selected rank statistics with severe *p*-value approximations version: 0.7–25) to calculate the optimal cut-off value of riskscore in both training and validation cohort. Based on the optimal cut-off value of riskscore, all patients were stratified into high-risk score group and low-risk score group. In order to be close to the clinical situation, patients with survival time less than 10 years were selected for the further analysis. Then, the ‘survival’ R package was used to analyze the differences in prognosis between the two groups, and the significance of prognostic differences between the two groups was evaluated using the log-rank test method. Next, the area under the ROC (AUC) corresponding for 1-, 3-, and 5-years were calculated to estimate the predictive accuracy of the signature. Further, we conducted univariate and multivariate Cox regression analyses to investigate whether the riskscore could serve as an independent prognostic factor for OSCC patients. Finally, a nomogram was established based on the riskscore and different clinical factors (including age, gender, grade and stage), and the calibration curve for 1-, 3-, and 5 years were plotted to assess the utility of the nomogram.

### Pathway enrichment analysis and mutation analysis

On the one hand, positive immunotherapy-related signatures were gathered from the known literature, and enrichment scores were quantified using ‘GSVA’ R package (Hu et al., 2021). On the other hand, hallmark gene set (https://www.gsea-msigdb.org/gsea/downloads.jsp) was also selected to employ correlation analysis with riskscore. In addition, gene set enrichment analysis (GSEA) was conducted using the ‘ClusterProfiler’ R package with curated gene sets, ontology gene sets and oncogenic signature gene sets as reference sets. Then, somatic mutation data of all tumors from cBioPortal database (https://www.cbioportal.org/datasets) were gathered to compare differences of tumor mutation burden (TMB) values between OSCC patients and patients with other tumors. Finally, genes with more than 10 mutations and *p* < 0.05 between the two groups were considered mutation-differential genes, and interaction effect analysis was performed among these mutation-differential genes using ‘maftools’ R package.

### Exploration of immune features and prediction for immunotherapy

Based on the expression profile, we used the ‘ssGSEA’ R package to calculate scores of 35 immune infiltrating signatures for each sample. Then, correlation analyses were performed between the expression of each signature lncRNA and 22 immune cells. Immune scores, stromal scores and estimate scores was calculated using ‘estimate’ R package. Further, differential expression analysis of 50 immune-checkpoint–relevant genes was conduct between the two groups. Importantly, immunotherapeutic response was predicted by tumor immune dysfunction and exclusion (TIDE) algorithms, that patients with TIDE score >0 were regarded not be susceptible to immunotherapy, and patients with TIDE score <0 were regarded be susceptible to immunotherapy (Jiang et al., 2018). Moreover, subclass mapping algorithm was applied to determine the appropriateness of patients between two risk groups for CTLA-4 inhibitor therapy or PD-1 inhibitor therapy (Hoshida et al., 2007). Finally, the pharmacy medicine response of subtype samples was also predicted based on the largest public pharmacogenomics database [Pharmaceutical Sensitivity Genomics in Cancer (GDSC), https://www.cancerrxgene.org/].

### Statistical analysis

All the R packages used were based on the R software (version 4.0.2). Statistical significance for comparisons between two groups was compared using Wilcox test, and continuous variables were compared using Wilcoxon rank-sum test. All *p* values were set as two-sided, and a *p* value < 0.05 was regarded as statistically significant.

## Results

### Data processing and identification of cuproptosis-related lncRNAs

**FIGURE 1:**
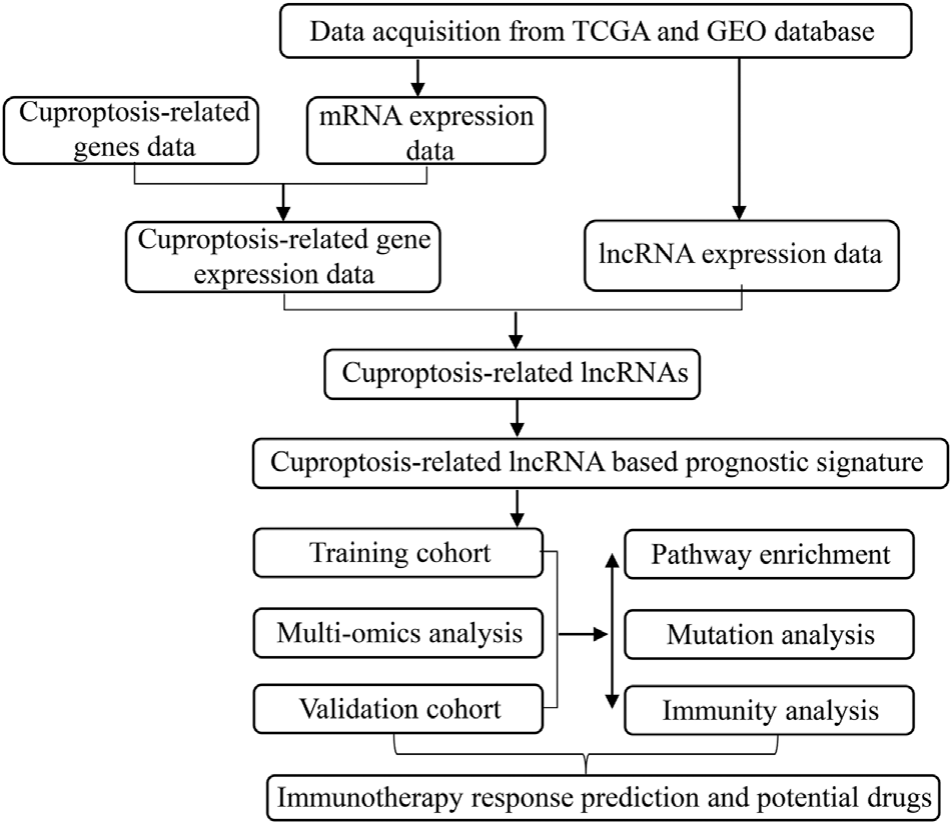
The flow chart of our study.

The flow chart of our study was shown in Figure 1. After normalizing the expression data and excluding clinical data with missing survival information, a total of 390 OSCC patients in TCGA were assigned into the training cohort and 206 OSCC patients in GSE42743 were assigned into the validation cohort. A total of 13 cuproptosis-related genes were collected from the known literature. Finally, based on co-expression analysis using pearson’s correlation algorithm with the criteria |Cor| > 0.5 and *p* < 0.001, 917 cuproptosis-related lncRNAs were identified and were listed in Supplementary Table S1.

### Cuproptosis-related lncRNA based prognostic signature

**FIGURE 2:**
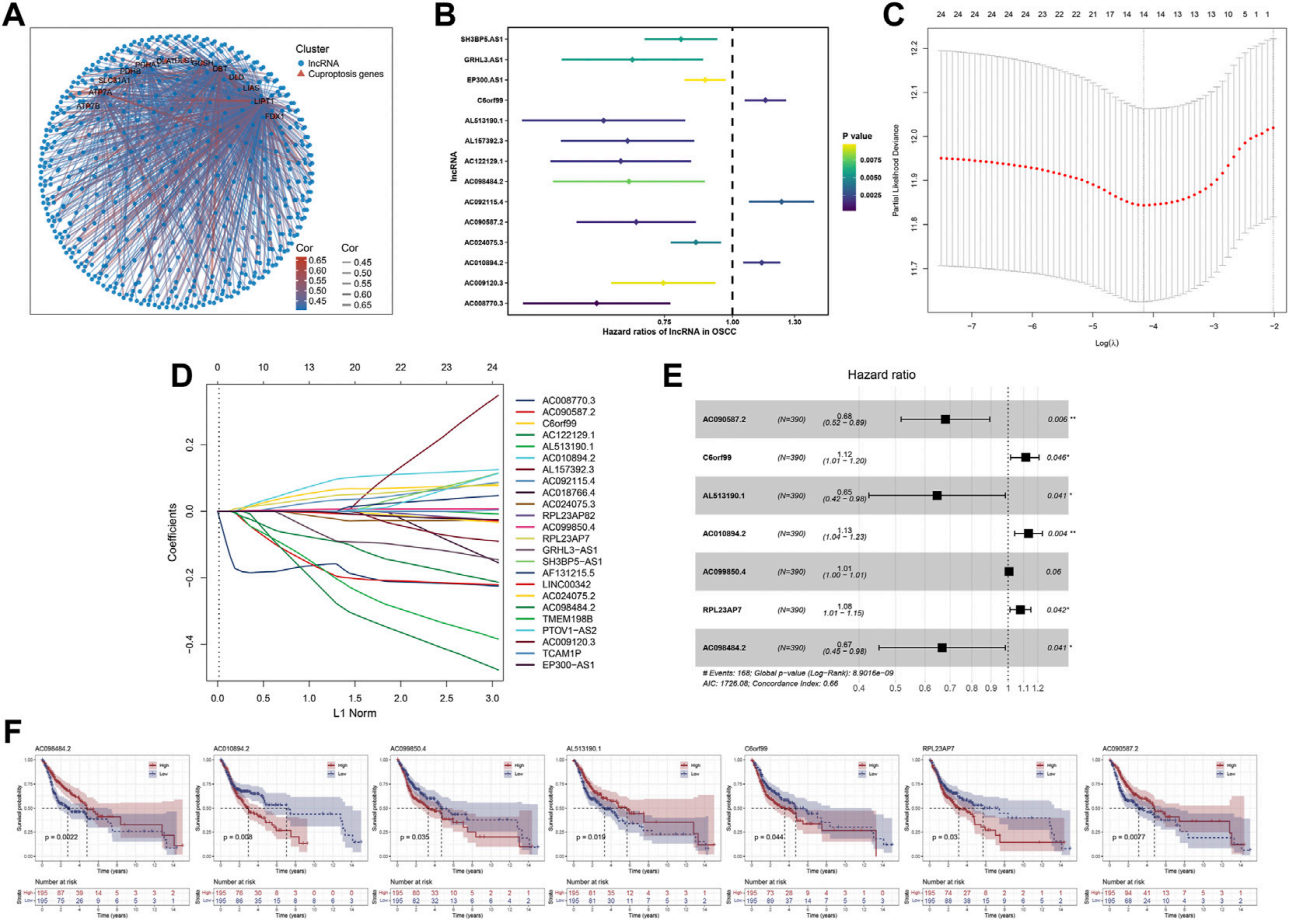
Identification of cuproptosis-related lncRNA based prognostic signature. Notes: **(A)** Correlation analysis between the expression of cuproptosis-related genes and the lncRNAs. **(B)** Univariate cox regression analysis of cuproptosis-related lncRNAs. **(C**,**D)** Lasso regression analysis screened 24 cuproptosis-related lncRNAs **(E)** Forest plot of 7 prognostic lncRNAs identified by multivariate cox regression analysis **(F)** Kaplan-Meier survival curves of signature lncRNAs in OSCC.

Figure 2A showed the results of the co-expression analysis between cuproptosis-related genes and lncRNAs. Then, univariate Cox regression proportional hazards analysis was performed on these cuproptosis-related lncRNAs, among which 24 lncRNAs associated with OS with *p* < 0.01 were screened (Supplementary Table S2), and Figure 2B showed the 14 lncRNAs with the lowest *p* value. Furthermore, 24 OS-related lncRNAs extracted were performed lasso cox regression analysis after 1,000 iterations for further selection (Figures 2C,D). As shown in Figure 2E, we constructed a prognostic signature consist of 7 cuproptosis-related lncRNAs in the training cohort including AC090587.2, C6orf99, AL513190.1, AC010894.2, AC099850.4, RPL23AP7, AC098484.2, and coefficients for each signature lncRNA were obtained using multivariate Cox regression analysis (Supplementary Table S3). For each prognostic lncRNA, we also conducted kaplan-meier (K-M) survival analysis to compare the OS time between the groups with high and low expression (Figure 2F).

### Evaluation and verification of the prognostic signature

**FIGURE 3:**
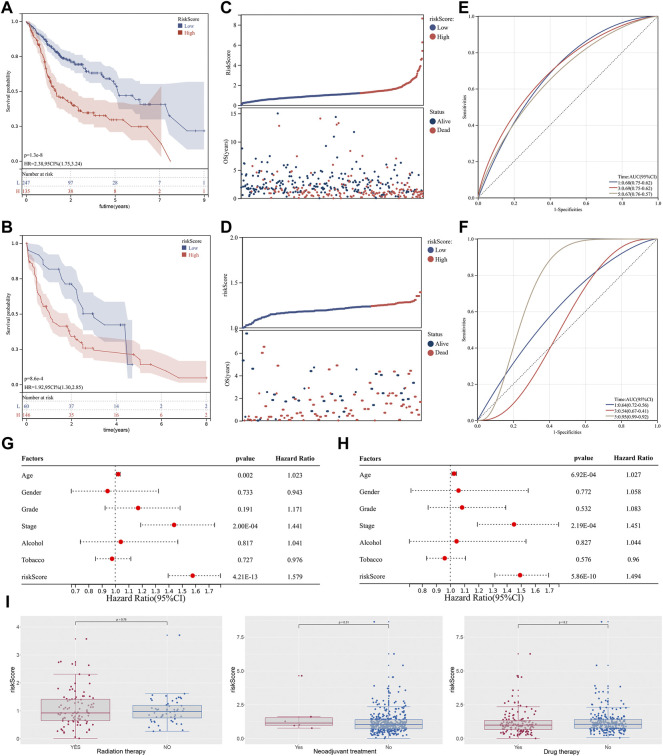
Identification of cuproptosis-related lncRNA based prognostic signature. Notes: **(A**,**B)** Kaplan-Meier curve analysis of the signature between two groups in both training cohort and validation cohort. **(C**,**D)** In the risk plot, the mortality rate of patients dramatically increased with an increase in riskcore. **(E**,**F)** Time-dependent ROC curve analysis of 1-,3- and 5 years in both training cohort and validation cohort. **(G**,**H)** Univariate and multivariate cox regression analysis of between the riskscore and clinical features of the signature. **(I)** Differential expression analysis of riskscore in patients with different treatments status.

Based on the ‘maxstat’ R package (Maximally selected rank statistics with several *p*-value parity Version: 0.7–25), the optimal cut-off values of riskscore calculated were 1.212793 in training group and 1.23567 in validation group. Based on this, patients were divided into high and low groups, and the ‘Survival’ R package was further used to analyze the difference in prognosis between the two groups. To compare prognosis between groups based on samples, the log-rank test was applied in patients with survival time less than 10 years, and a significant difference in prognosis was finally observed in both training cohort (Figure 3A; HR = 2.38 (1.75–3.24), *p* = 1.3e−08) and validation cohort (Figure 3B; HR = 1.92, *p* = 8.6e−e), indicating that riskscore may predict the prognosis of OSCC patients. In tandem with the increase in riskcore, the mortality rate of patients also increased dramatically both in training cohort (Figure 3C) and validation cohort (Figure 3D). Furthermore, the time-dependent AUC values were calculated to assess the predictive sensitivity and specificity of the signature. Results showed that the AUC value corresponding for 1-, 3-, and 5-years were 0.68, 0.69, and 0.69 in the training cohort (Figure 3E) and 0.64, 0.54, and 0.95 in the validation cohort (Figure 3F). In addition, univariate and multivariate Cox regression analyses were performed to investigate whether the riskscore could serve as an independent prognostic factor. Univariate Cox regression showed that stage (*p* = 7.85e−05) and riskscore (*p* = 1.21e−13) were associated with the prognosis (Figure 3G). However, the multivariate Cox regression analysis revealed that only the riskscore rather than other clinical factors such as alcoholism and smoking remained being predictive for the prognosis (Figure 3H). More remarkably, we found that no difference in riskscore between patients in the treated and untreated groups, suggesting that riskscore was independent of whether patients have received treatments (Figure 3I).

### Development of the riskscore based nomogram

**FIGURE 4:**
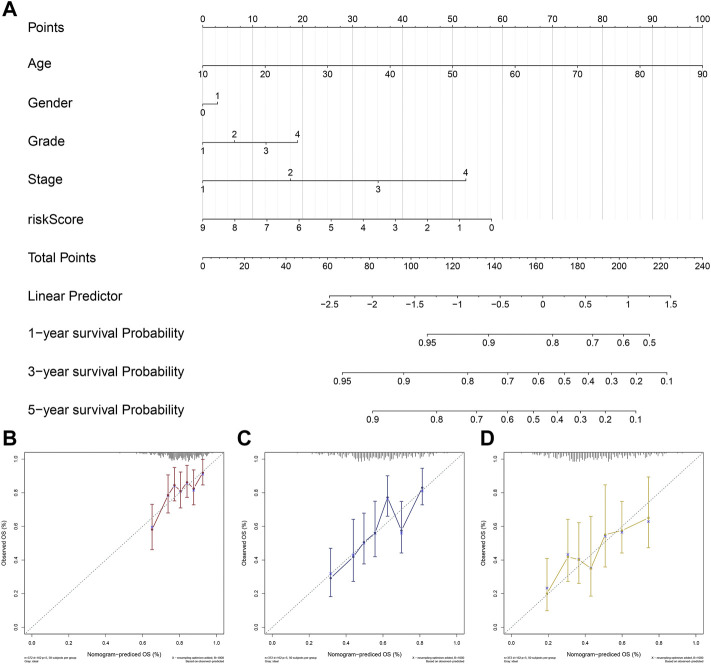
Development and Validation of a riskscore based nomogram. Notes: **(A)** A riskscore based nomogram was constructed based on riskscore and clinical characteristics of OSCC. **(B–D)** Calibration curves of the nomogram for the estimation of survival rates at 1-**(B)**, 3-**(C)** and 5 years**(D)**.

Based on ‘rms’ and ‘nomogramEx’ R packages, a riskscore-based nomogram was established with other clinical factors including age, gender, grade, stage (Figure 4A). C-index was calculated using bootstrap method with 1000 resamples to assess the utility of the nomogram, from which we obtained the C-index of the nomogram was 0.635. In addition, calibration curves for predicting the probability of 1-, 3- and 5-years for OSCC patients were plotted (Figures 4B–D).

### Pathway enrichment and tumor mutation burden

**FIGURE 5:**
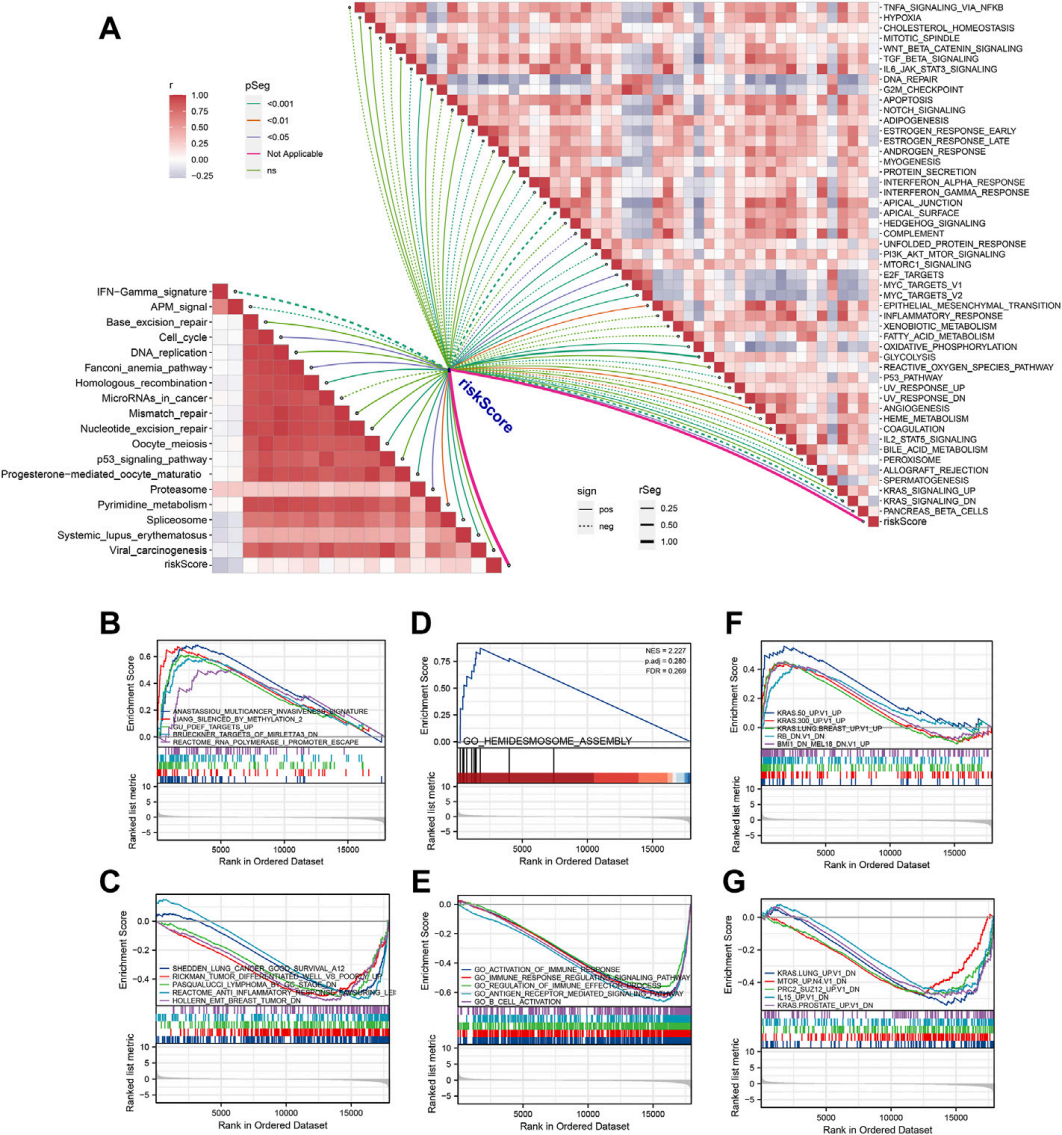
Pathway enrichment analysis of the signature. **(A)** Correlation analysis between riskscore and the enrichment scores of immunotherapy-predicted pathways as well as hallmark gene signatures. **(B**,**C)** Using curated gene sets, GSEA analysis was performed between two risk groups. **(D**,**E)** Using ontology gene sets, GSEA analysis was performed between two risk groups. **(F**, **G)** Using oncogenic signature gene sets, GSEA analysis was performed between two risk groups.

**FIGURE 6:**
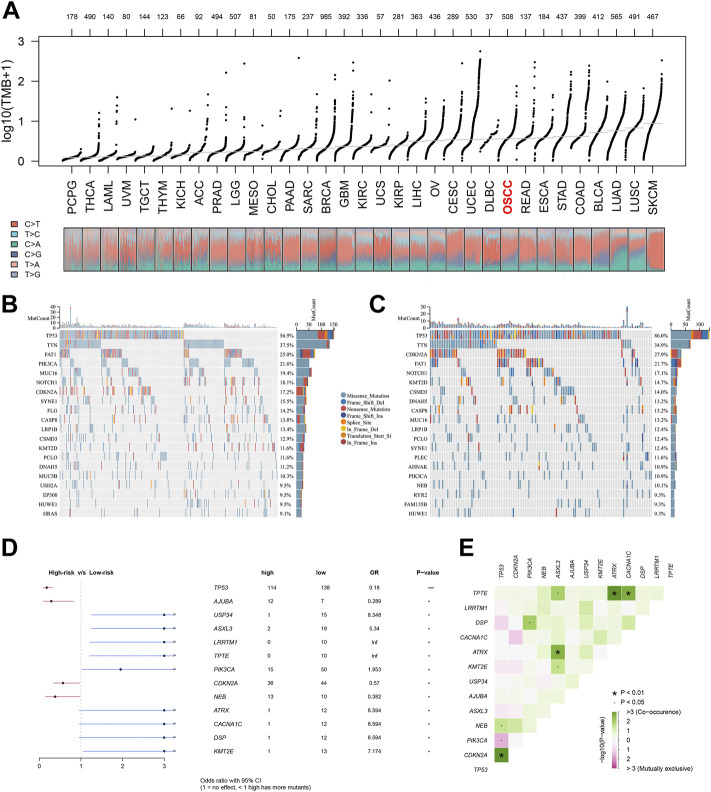
Tumor mutational analysis of the signature. Notes: **(A)** Distribution of TMB values of all tumors in TCGA database. **(B**, **C)** Mutational landscapes of both high-risk group and low-risk groups. **(D**, **E)** Forest plot of differentially mutated genes in patients between two risk groups.

Each patient was assigned an enrichment score based on the known immunotherapy-related signatures and hallmark gene set using ‘GSVA’ R package. Then the result of correlation analysis between these signatures and riskscore showed that the riskscore was positively correlated with almost all of these immunotherapy-related positive signatures (Figure 5A). To further clarify the roles of biological processes and pathways in OSCC patient prognosis, we choose curated gene sets, ontology gene sets and oncogenic signature gene sets as reference sets to conduct GSEA analysis between two groups (Figures 5B–G). Meanwhile, somatic mutation data of all tumors from TCGA were gathered and we found that level of TMB value in OSCC patients was relatively higher compared with other most tumors (Figure 6A). Furthermore, the mutational landscapes of both high-risk group and low-risk groups were visualized, from which we can observe that patients in both high-risk group and low-risk groups were prone to TP53, TTN, FAT1 and NOTCH1 mutations (Figures 6B,C). Moreover, genes with more than 10 mutations and *p* < 0.05 between the two groups were considered differentially mutated genes. Analysis of mutation difference between two risk groups was performed, and results revealed that USP34, ASXL3, LRRTM1, TPTE, PIK3CA, ATRX, CACNA1C, DSP and KMT2E were differentially mutated genes in low-risk group while TP53, AJUBA, CDKN2A and NEB were differentially mutated genes in high-risk group (Figure 6D). In addition, interaction effects were observed among mutations of these genes (Figure 6E).

### Immunity exploration and immunotherapy response prediction

**FIGURE 7:**
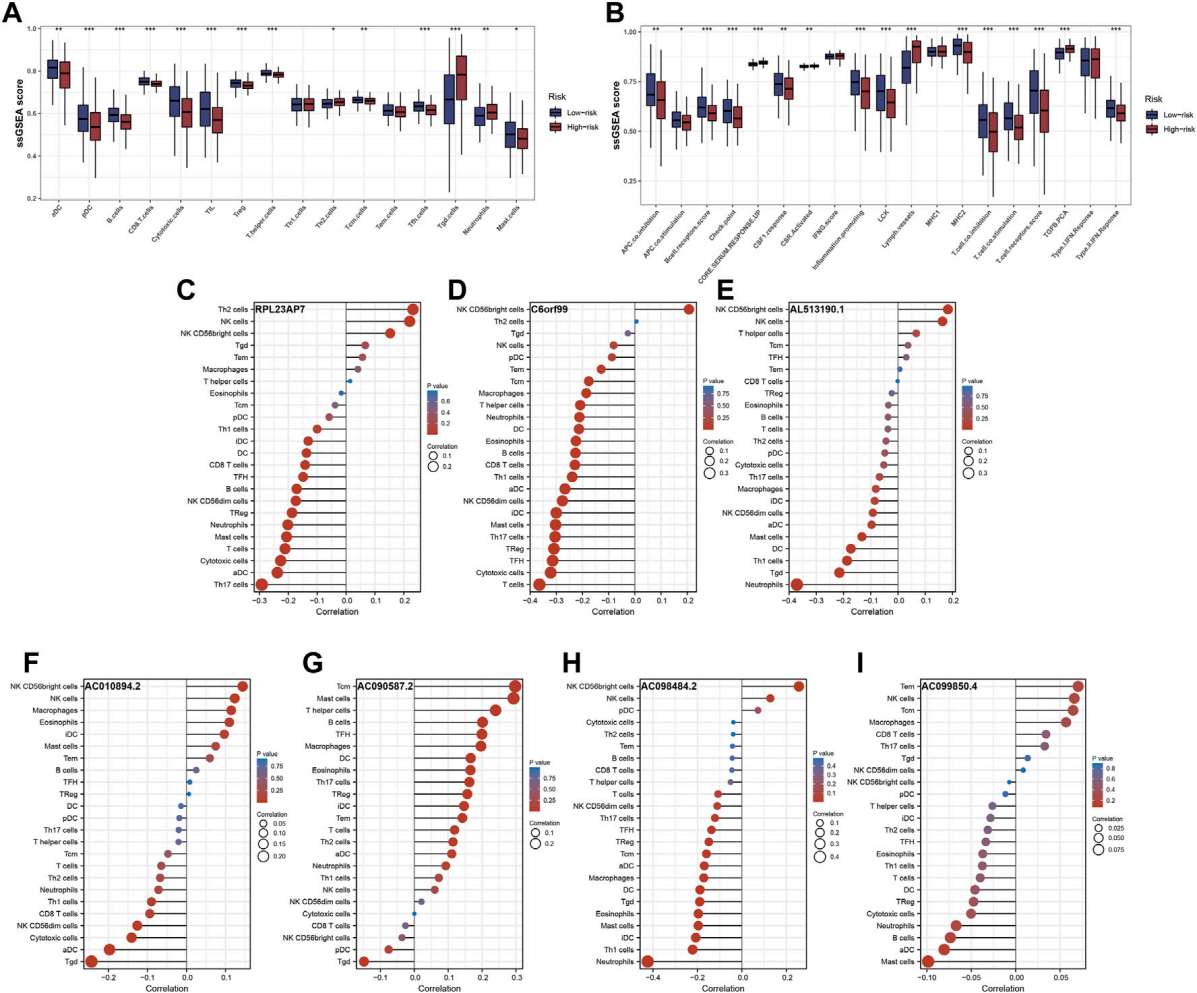
Immune infiltrating signatures of the signature. Notes: **(A**,**B)** Differential expression analysis of immune cells and immune functions between the two groups. **(C**–**I)** Correlation analysis between the expression of each signature lncRNA and 22 immune cells.

**FIGURE 8:**
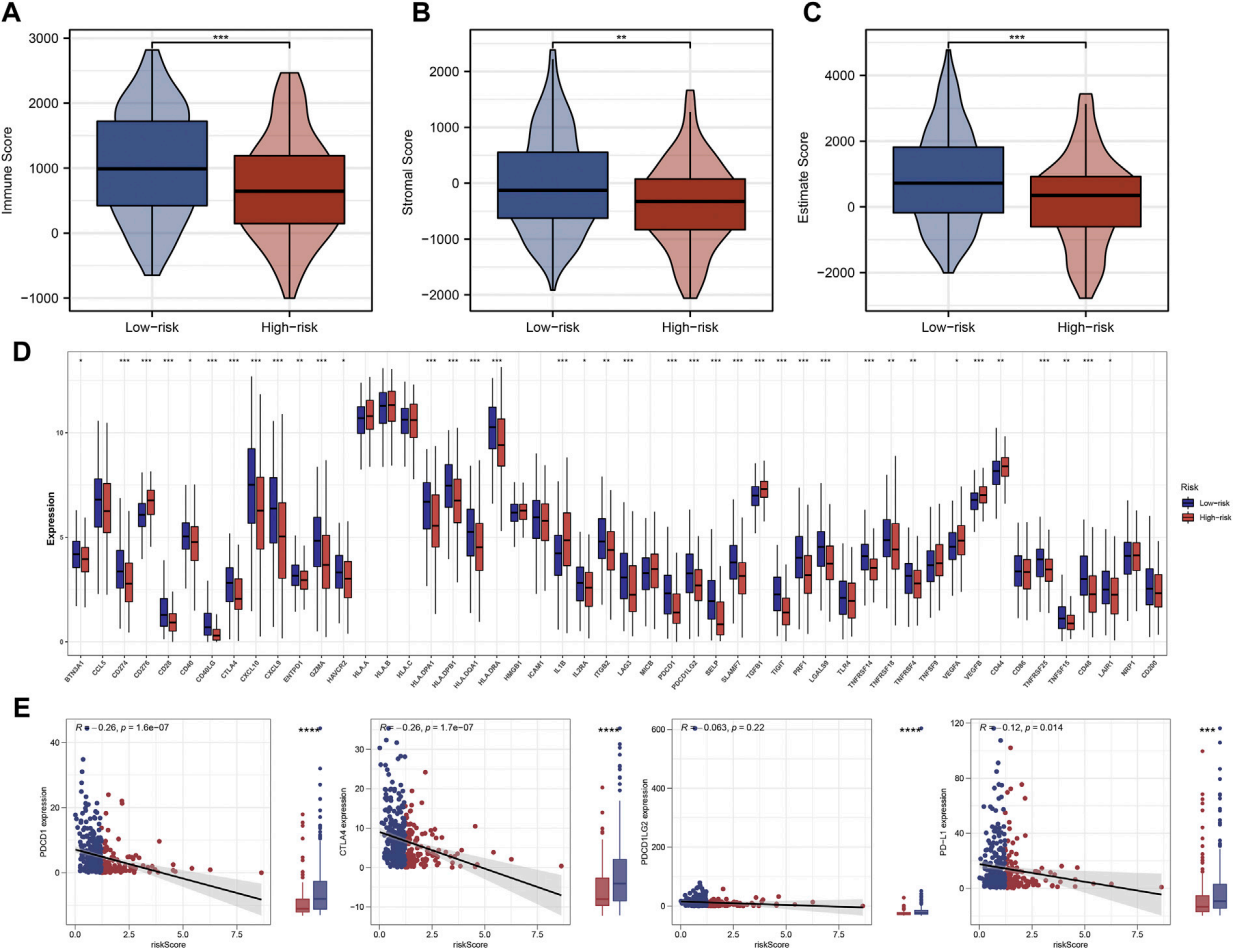
Significant difference of immunity features between two groups. Notes: **(A**–**C)** Patients with low riskscore had higher immune scores, stromal scores and estimate scores compared with patients with high riskscore. **(D)** Differences in expression of 50 common immune-checkpoint–relevant genes between two the groups. **(E)** Correlations analysis showed that riskscore negatively correlated with CTLA4 and PD-1 expression.

**FIGURE 9:**
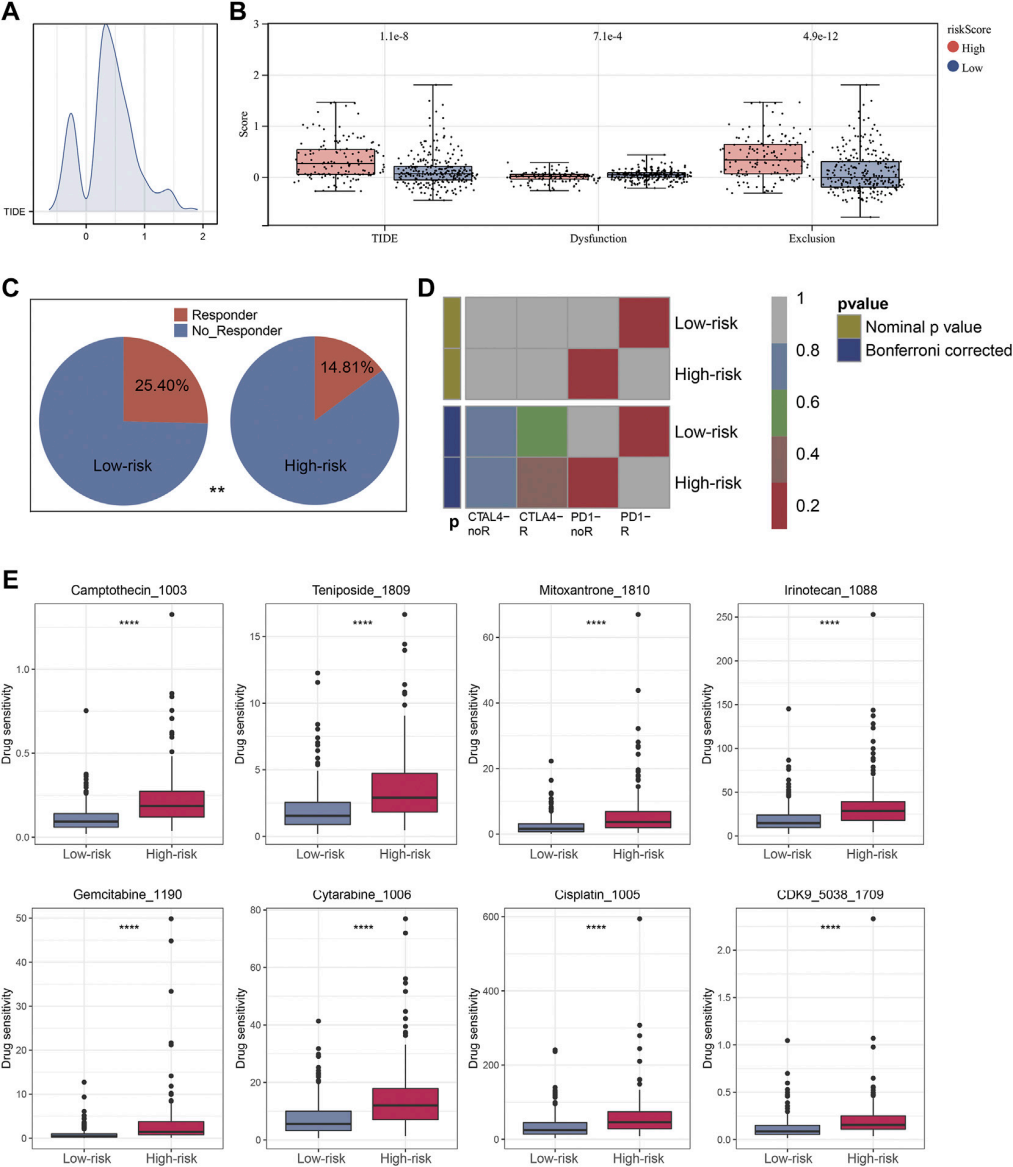
Immunotherapeutic response prediction and screening of potential drugs. Notes: **(A)** Distribution of TIDE score of each OSCC patient. **(B)** Patients in low risk group have a lower level of TIDE score, Dysfunction score and Exclusion score. **(C)** Immunotherapy has a higher success rate with low-risk patients. **(D)** The subclass mapping analysis showed that low-risk patients were more likely to benefit from PD-1 inhibitor therapy. **(E)** The top eight drugs with the maximum log2FC values and the minimum *p* values in GDSC database.

The ‘ssGSEA’ R package was firstly performed to quantify scores of 35 immune infiltrating signatures including immune cells and immune functions for each patient, from which significant differences were observed between the two groups (Figures 7A,B). Then correlation analyses were also conducted between the expression of each signature lncRNA and 22 immune cells (Figures 7C–I). Meanwhile, the ‘ESTIMATE’ R package was performed to calculate the immune scores, stromal scores and estimate scores for each patient, and we all these scores were higher in patients with low riskscore compared with patients with high riskscore (Figures 8A–C). Given that immune checkpoint inhibitor therapy has shown important clinical advances in different tumors, the distribution of 50 immune-checkpoint–relevant genes between the two groups was presented in Figure 8D. In addition, analysis of correlations revealed that riskscore correlated negatively with CTLA4 and PD-1 expression (Figure 8E). Notably, tumor immune dysfunction and exclusion, a novel algorithm used to predict the likelihood of response to immunotherapy, was performed to explore the association between the risk stratifications and the effect of immunotherapy. The distribution of TIDE scores in OSCC patient was shown in Figure 9A. Following, the results of TIDE analysis showed that patients with low riskscore had a lower TIDE score and Exclusion score, suggesting that patients with low riskscore may be more susceptible to immunotherapy (Figure 9B). And we can see that there were 25.40% patients with low riskscore responded to immunotherapy while only 14.81% patients with high riskscore responded to immunotherapy (Figure 9C). To verify our results, subclass mapping analysis was also performed to determine the appropriateness of patients between two risk groups for immunotherapy. As expected, PD-1 checkpoint therapy has been shown to be more beneficial for patients with low riskscore (Figure 9D). Finally, based on GDSC database, we calculated the IC50 of 179 drugs to identify drugs whose sensitivity differs between two risk groups using R ‘oncoPredict’ package (Supplementary Table S4), and the top eight drugs with the most significant sensitivity differences were shown in Figure 9E.

## Disscussion

Globally, well known for the high likelihood of progression and metastasis, head and neck tumors still pose the greatest risk of death from OSCC (Sieviläinen et al., 2019). In one hand, while diagnostic and therapeutic advances have made OSCC more detectable, the 5-year survival rate still remains at about 40–50% (Kumar et al., 2016). In another hand, after surgery, oral squamous cell carcinoma commonly recurs or invades the oral cavity because of its anatomical structure, that seriously affects the clinical outcomes of OSCC patients (Wong and Wiesenfeld, 2018). Recently, research revealed that in addition to dysregulate copper homeostasis triggering cytotoxicity, altered intracellular copper levels may affect cancer development and progression (Babak and Ahn, 2021). Meanwhile, a novel cell death pathway defined as cuproptosis has been proven can cause toxic protein stress and cell death by binding copper with lipoylated components of the tricarboxylic acid (TCA) cycle (Tsvetkov et al., 2022). By constructing the 4NQO oral carcinogenesis model, a research found a significant metabolic transformation characterized by an increase in glycolysis and a shortfall in the TCA cycle (Ge et al., 2021). In addition, accumulating evidence showed that the prognosis in patients with OSCC are significantly correlated with lncRNA molecular subtype. Our study firstly developed and validated a novel cuproptosis-related lncRNA based signature that can effectively indicate the prognosis of OSCC patients and immunotherapy response.

In this study, data from TCGA database was chosen as the training cohort and data from GSE42743 was chosen as the verification group. We first identified 917 cuproptosis-related lncRNAs on the basis of co-expression analysis. Using the univariate cox analysis, 24 cuproptosis-related lncRNAs linked closely to prognoses of OSCC patients were identified. Then a prognostic signature consists of 7 lncRNAs including AC090587.2, C6orf99, AL513190.1, AC010894.2, AC099850.4, RPL23AP7, AC098484.2 was constructed. Accurately predicting HNSCC outcomes and developing new therapeutic targets can be achieved with AC090587.2 and AL513190.1 (Zhou et al., 2022). There is evidence that C6orf99 is involved in diverse biological processes including spermatogenesis and development of spermatogonia that plays a key role in male infertility (Omolaoye et al., 2022). Moreover, the prognostic prediction of patients with HNSCC may also be affected by AC010894.2, which may serve as a potential therapeutic target (Lu et al., 2022). In addition, a study revealed that AC099850.4 may serve an important role in the tumorigenesis and progression of hepatocellular carcinoma (Qu et al., 2022). And there were few literatures has been reported about the other 2 lncRNAs. Based on the optimal cut-off values of riskscore calculated by the ‘maxstat’ R package, all OSCC patients were classified into high-risk group and low-risk group. Furthermore, the results from risk analyses, survival analyses, and 1-, 3-, and 5-year time-dependent ROC analyses between two risk groups well supported the effectiveness of the signature. And univariate and multivariate Cox regression analyses between riskscore and different clinical factors also revealed that the riskscore could serve as an independent prognostic factor for OSCC patients. Next, based on the risk score and other clinical factors, we developed a nomogram for clinicians, and in 3-, 5-, and 8-year calibration analyses, the nomogram could provide individualized, accurate survival prediction results.

Through GSEA and mutation burden analysis, we delved further into the underlying biological difference between the two groups. The results of correlation analysis showed that the riskscore were positively correlated with most immunotherapy-related pathways. In the meantime, we choose curated gene sets, ontology gene sets and oncogenic signature gene sets as reference sets to conduct GSEA analysis, and results suggested that most immune-related pathways were mainly enriched in patients with low riskscore. In addition, we found that patients in both high-risk group and low-risk groups were prone to TP53, TTN, FAT1 and NOTCH1 mutations, and USP34, ASXL3, LRRTM1, TPTE, PIK3CA, ATRX, CACNA1C, DSP and KMT2E were differentially mutated genes in low-risk group while TP53, AJUBA, CDKN2A and NEB were differentially mutated genes in high-risk group.

Importantly, we analyzed the landscape of immune cells and related immune function pathways between two groups, and we found that the expression immune infiltrating signatures were higher in patients with low riskscore. We also analyzed the difference of expression of 50 immune-checkpoint–relevant genes between the two groups. The results showed that patients in low risk group had a higher expression of most checkpoint–relevant genes, including PDCD1 and CTLA4, which has been reported as a predictive biomarker in cancer immunotherapy (Patel and Kurzrock, 2015). In addition, TIDE analysis showed that patients in low risk group were more susceptible to immunotherapy than patients in high risk group. Correspondingly, the same results were confirmed in subclass mapping algorithm, which demonstrated that patients in low risk group rather than in high risk group were more likely to benefit from PD-1 checkpoint therapy.

In the present work, a cuproptosis-related lncRNA based prognostic signature was successfully constructed and validated with superior predictive precision of prognosis and therapy for patients with OSCC. However, there were still several limitations in our research. Firstly, since our study only included individuals from Western populations, our study may have some population and genomic bias. Secondly, our prognostic signature was validated in only GSE42743 data. Though we identified some novel lncRNAs related to cuproptosis that have not been previously reported in OSCC, which may serve as a critical reference for later research, our work is an exploratory analysis for the lack of other external cohorts including the signature lncRNA expression data to validate our findings. Finally, further functional experiments need to be performed to investigate the potential molecular mechanisms between cuproptosis-related lncRNAs and the signature.

## Conclusion

In conclusion, we systematically performed bioinformatics analysis to explore the biological functions and prognostic value of cuproptosis-related lncRNAs in OSCC patients. We constructed and validated a novel cuproptosis-related lncRNA based prognostic signature, and possible immune-related mechanism underlies this signature were identified. Lastly, and most importantly, all the results in our study indicated that patients with low riskscore were more susceptible to immunotherapy, especially PD-1 inhibitor therapy.

## Data Availability

The datasets presented in this study can be found in online repositories. The names of the repository/repositories and accession number(s) can be found in the article/Supplementary Material.
